# RANK/RANKL/OPG Signaling in the Brain: A Systematic Review of the Literature

**DOI:** 10.3389/fneur.2020.590480

**Published:** 2020-11-19

**Authors:** Anton Glasnović, Niall O'Mara, Nataša Kovačić, Danka Grčević, Srećko Gajović

**Affiliations:** ^1^Department of Histology and Embryology, Zagreb University School of Medicine, Zagreb, Croatia; ^2^Croatian Institute for Brain Research, Zagreb University School of Medicine, Zagreb, Croatia; ^3^Department of Medicine, Cork University Hospital, Cork, Ireland; ^4^Department of Anatomy, Zagreb University School of Medicine, Zagreb, Croatia; ^5^Department of Physiology and Immunology, Zagreb University School of Medicine, Zagreb, Croatia

**Keywords:** OPG - RANKL - RANK, BBB rupturing, neural mediators, stroke repair, multiple sclerosis - etiology, neuroinflammatory cytokines

## Abstract

Together with its dominant immunological and bone remodeling involvement, RRO axis, comprising of receptor activator of nuclear factor-κB (RANK), RANK ligand (RANKL), and osteoprotegerin (OPG) signaling, is as well-implicated in CNS functioning and corresponding pathologies. The CNS aspects of RANKL/RANK/OPG (RRO) axis were systematically reviewed. With search 10 databases, and 7 additional resources from first article publication to July 2019, resulted in total 2,222 hits, from which 33 relevant articles were selected. The elements of RRO axis in CNS include cells involved in neuroinflammation, predominantly in microglia, but as well in resident macrophages and inflammatory cells migrating across the blood-brain barrier. The expression in neurons and oligodendrocytes is mainly confined to processes of differentiation and cell death. RRO axis tunes the neuroinflammatory response, depending on the molecular, cellular and pathological context. RANK/RANKL signaling is neuroprotective in TLR-mediated inflammation, while OPG seems detrimental in stroke, but beneficial in multiple sclerosis. The levels of RRO axis elements can serve as biomarkers in the blood and cerebrospinal fluid. They act as neuroprotectant after brain damage even being implicated in body weight- and thermo-regulation. As derivatives of RRO axis already exist as therapeutic agents in bone remodeling, it would be intriquing to see if these or new RRO-based pharmaceuticals would appear effective in CNS therapies.

## Introduction

The receptor activator of nuclear factor-κB (RANK)/RANK ligand (RANKL)/osteoprotegerin (OPG) (RRO axis) was originally discovered through parallel investigations in the late 1990's within the immune and bone systems (1–4). This signaling triad regulates a variety of metabolic and cellular processes, thus its disturbance contributes to the pathogenesis of bone and immune diseases, such as rheumatoid arthritis, osteoporosis, diabetes mellitus and certain types of cancer. Recent evidence suggests that the triad is also implicated in a number of neurological conditions, in particular in various aspects of neural tissue damage and subsequent reparative processes (5). Further elucidation of the functional relationship between certain neurological diseases and the disturbances in the RRO axis may provide potential diagnostic and therapeutic targets.

The RRO axis involves the interactions between three members of the tumor necrosis factor (TNF) superfamily (TNFSF), RANK (also known as TNFRSF11a), its cognate ligand RANKL (also known as TNFSF11) and a decoy receptor OPG (also known as TNFRSF11b). RANKL, a member of the TNF ligand superfamily binds to RANK and a decoy receptor OPG, both from the TNF receptor (TNFR) superfamily (6). RANKL, secreted by T cells, has been shown to enhance the immune response by promotion of dendritic cell survival and function mediated by RANK signaling (2, 7). Conversely, RANKL may induce immune tolerance by stimulating the regulatory T cell (Treg) differentiation in certain autoimmune diseases such as diabetes mellitus and chronic colitis (8, 9). Based on these results, it can be concluded that, depending on the other microenvironmental signals, the RRO system may either activate or suppress immune response.

In addition to the immune system, the RRO axis is well-characterized in the context of bone remodeling. RANKL, secreted by osteoblasts, osteocytes, hypertrophying chondrocytes, and bone marrow stromal cells, stimulates the responsive RANK-bearing osteoclast precursors, to differentiate into active bone-resorbing osteoclasts. This negative feedback-loop is important for physiological bone remodeling, and the equilibrium between bone formation and bone resorption. OPG is secreted by osteoblasts, bone marrow stromal cells, B cells, and dendritic cells. It acts as a decoy receptor for RANKL, preventing its interaction with RANK and, consequently, blocking osteoclast maturation Moreover, the imbalance in RANKL/RANK signaling may lead to a number of bone disorders, such as rheumatoid, arthritis and osteoporosis (10–13).

Members of the RRO axis are widely expressed in different embryonic and adult tissues (5, 14). RANK is mostly expressed by monocyte/macrophage lineage cells. Depending on the milieu created by the mediators in the residing tissue, these cells may be directed toward effector myeloid progenies or remain dormant until receiving maturation signals (15, 16). In addition to classical pro-inflammatory mediators, activated T and B cells produce RANKL during inflammatory and immune responses. It is important to acknowledge that there are two forms of RANKL, one membrane-bound, and the other one soluble, which is detached from the surface of the cell by proteases. Lymphocytes serve as a significant source of soluble (s)RANKL, which potentiates dendritic cell activation and osteoclast differentiation (17–19). In particular, helper T (Th) cells, Th1 and Th17, are the primary T cell subsets associated with immune activation and subsequent stimulation of osteoclastogenesis. Conversely, Tregs suppress immune response, and inhibit osteoclast differentiation (20, 21). In addition to the activation of myeloid cells, it is hypothesized that RANKL further potentiates inflammatory processes through the provision of bone-derived Ca^2+^ ions, which interact with the calcium sensing receptors and simulate proinflammatory mediators (20, 22). Such pathogenic roles of RRO axis have been observed in states of pathological or persistent inflammation where reduction in bone mass has been associated with immune cell activation (21, 23–25).

Together with the role in bone and immune system of RRO axis, the growing evidence implicates that it also has role in nervous system, therefore this review focuses to this particular aspect. The purpose of this article is to provide a systematic review on the current knowledge of the RRO axis in relation to the neurological diseases.

## Search Methods for Identification of Studies

We conducted a comprehensive search (from the first published article available online to July 2019) and developed detailed search strategies, based on the strategy for MEDLINE (using MeSH terms and text keywords) but revised appropriately for each resource, and used English language while searching the entire database.

We searched the following databases:

1. Cochrane Central Register of Controlled Trials (CENTRAL/Cochrane Library; via Ovid)
2. MEDLINE (via Ovid)
3. Scopus
4. Web of Science Core Collection (via Web of Science)
5. Current Contents (via Web of Science)
6. SciELO Citation Index (via Web of Science)
7. KCI-Korean Journal Database (via Web of Science)
8. Russian Science Citation Index (via Web of Science)
9. BIOSIS Citation Index (via Web of Science)
10. Data Citation Index (via Web of Science).

We searched additional resources including:

1. ProQuest Dissertations & Theses Global
2. OpenGrey
3. African Index Medicus
4. IndMED
5. ClinicalTrials.gov
6. WHO International Clinical Trials Registry Platform (WHO ICTRP)
7. CenterWatch.

**Figure 1:**
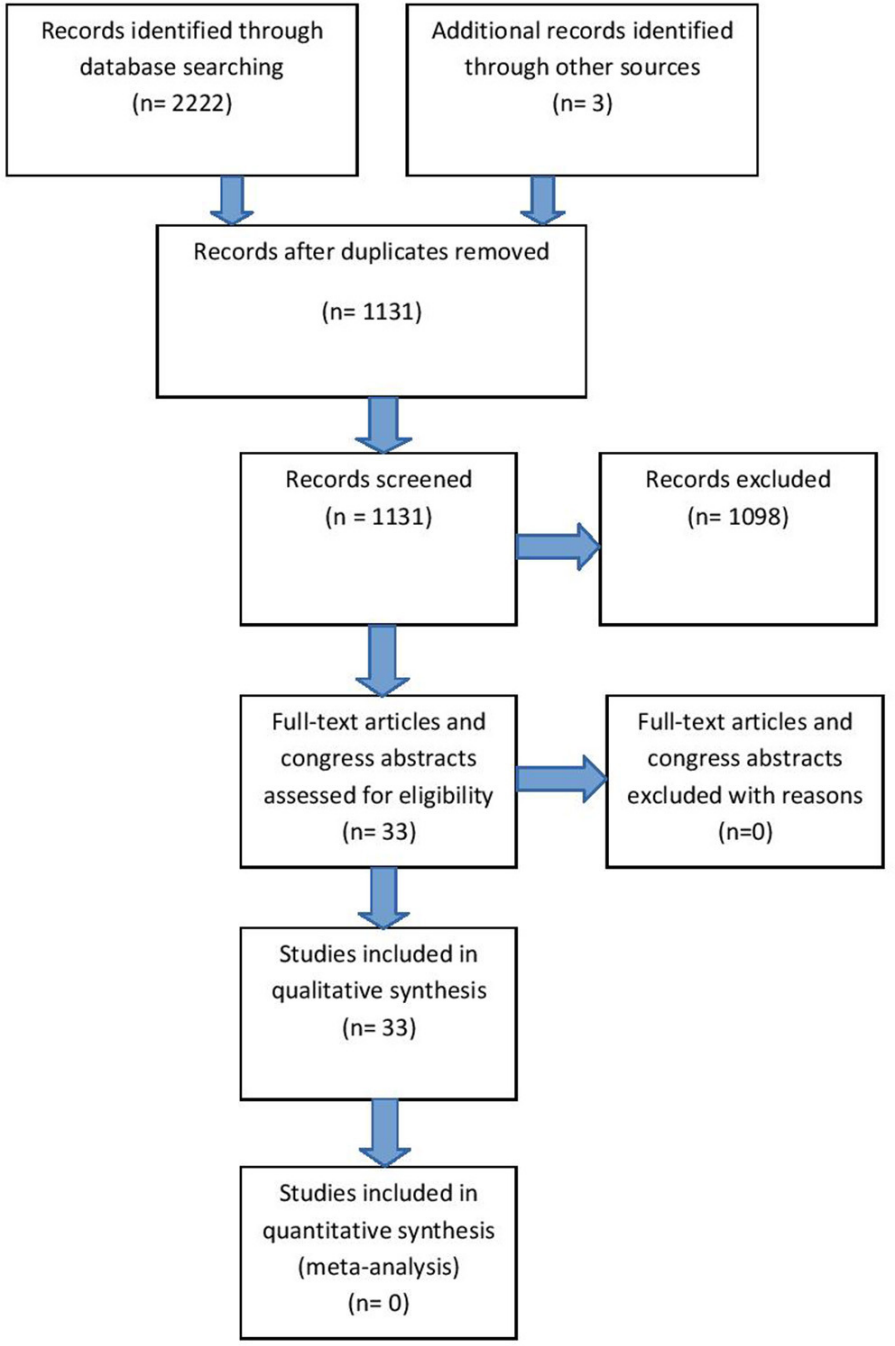
Study flow diagram (From: The PRISMA Group 2009).

Our search strategy is described in detail in Supplemental Material 1, resulting in 33 references for qualitative synthesis (Figure 1, Supplemental Material 2). All included references had to be relevant and eligible for the major topic of our research and that was RANKL/RANK/OPG axis in CNS. All studies, including research articles, case reports, experimental studies, conferrence abstracts and preliminary results were comprised in the review. Exclusion of studies was based upon non-relevance to any of the given major topic (not pertaining to central nervous system and RANKL/RANK/OPG axis). Namely, we excluded studies that used the same abbreviation for different categories (e.g., OPG being optical pathway glioma), assessed RRO axis in systemic vascular diseases, or included inflammatory/autoimmune diseases with minor reference to CNS tissues. Finally, 1,098 studies were excluded for consideration from our review. Critical appraisal of selected studies was done by all authors.

Relatively low number of references on this topic (33 in total), due to the risk of bias, may indicate an insufficient number of studies whose results could directly and undoubtedly be used for further research or some other application in practice.

## Cellular and Soluble Elements of RRO Axis in the Brain

The RRO axis and osteoclast paradigm is applicable to microglial cells as both osteoclasts and microglia share some developmental and functional properties, including hematopoietic origin, phagocytosis, and immunomodulation (26). Moreover, this can be extended to all myeloid populations within the central nervous system (CNS), including parenchymal microglia in the nerve tissue, perivascular phagocytic cells, meningeal macrophages, and choroid plexus macrophages (27–30). In contrast to other tissue-resident macrophages which arise from the embryonic yolk sac and fetal liver, microglia originates exclusively from yolk sac-derived hematopoietic progenitors (27). These progenitors infiltrate the brain during early development, differentiate into microglia, and maintain their population by self-renewal (26).

Recent studies have demonstrated that members of the RRO axis are expressed by several types of cells within neural tissue; this includes microglia, other resident macrophages, neurons and oligodendrocyte precursor cells (28–30). It has also been noted that RANKL, alongside brain-derived neurotrophic factor (BDNF) could induce differentiation of human umbilical cord blood cells into neurons and glial cells, and that both have synergistic effect (31). Moreover, the expression pattern of RRO axis is tuned by neuro-inflammatory events associated with blood-brain barrier (BBB) disruption. Inflammatory monocytes cross the BBB by the stepwise process initiated by various stimuli, such as soluble mediators secreted by pro-inflammatory T cells and activated microglia, becoming infiltrating macrophages. In case of further BBB damage, additional cells passively enter and accumulate at the inflammatory site within CNS (32). In the early phase of the inflammation, RRO mediators are produced almost exclusively by microglia, whereas in the later phases of the inflammatory response they originate from mixed microglia/macrophage (M/M) cells (32–34).

*In vitro* studies showed that, in certain conditions of cellular stress, OPG can be expressed by neurons and oligodendrocyte progenitors, protecting the neurons from apoptosis caused by death receptor stimulation (35). However, this effect and its consequences *in vivo* are still to be confirmed.

## RRO Axis in Context of Microglial Regulation

**Figure 2:**
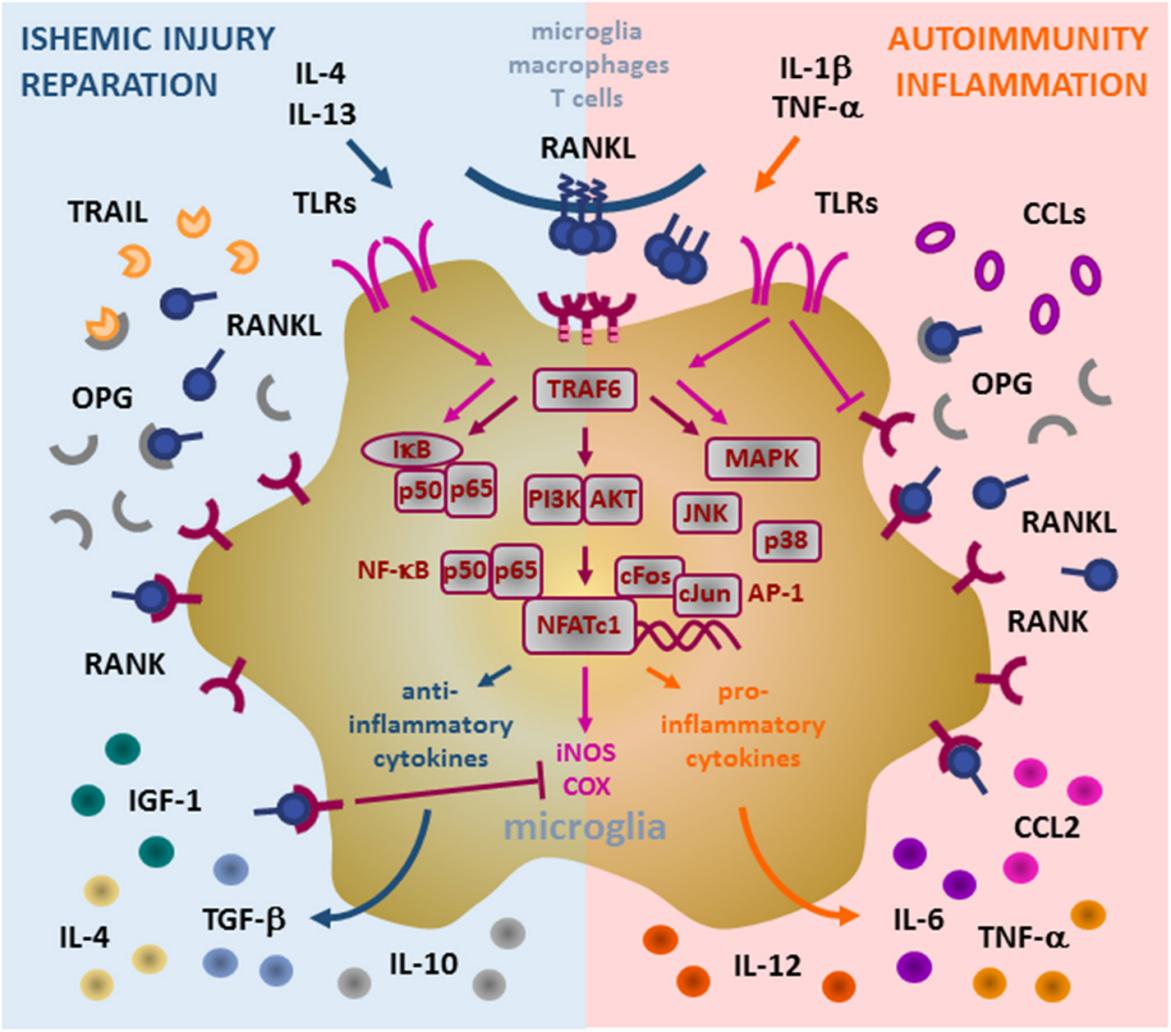
Role of RANK/RANKL/OPG (RRO) axis in microglia. Binding of membrane or soluble RANKL induces trimerization of RANK expressed on the microglia and initiates signal transduction. RANK lacks the intrinsic enzymatic activity in its intracellular domain and transduces a signal by recruiting adaptor molecules from the TRAF family of proteins, mainly TRAF6 (via three TRAF6-binding sites in its C-terminal cytoplasmic tail). Upon formation of the RANK/TRAF6 complex, multiple signaling pathways are activated, including NF-κB (activation of p65:p50 subunits/IκB complex), PI3K/AKT, and MAPK (including p38 and JNK). These signaling cascades potently induce transcription factors NFATc1 and AP-1 (cFos and cJun) to initiate transcription of target genes. In pathological conditions, microglia acquire different context-dependent functions. Under ischemic conditions, induced by anti-inflammatory IL-4 and IL-13 cytokines, microglia may exhibit neuroprotective action by production of immunosuppressive and reparative cytokines and growth factors (IL-4, IL-10, TGF-β, and IGF-1). Under such response, induced RANKL production and overexpression of RANK contribute to transcription of anti-inflammatory mediators and suppress iNOS and COX induced by TLR (TLR3/TLR4) signaling, whereas upregulation of OPG blocks ligation of TRAIL and protects microglia from apoptosis. During inflammatory and autoimmune processes, pro-inflammatory IL-1beta and TNF-alpha cytokines drive microglia to support inflammatory reaction. Enhanced RANKL/RANK signaling in this context may promote up-regulation of pro-inflammatory cytokines and chemokines (including TNF-α, IL-6, CCL2, IL-1β), which in turn attract additional inflammatory and immune cells, induce Th1 polarization of infiltrating T cells by IL-12 and thus, neural tissue damage. TLR signaling interfere with RANK signaling in microglia, namely TLR4 suppress RANK expression but auto-amplify TLR3-initiated cascades (NK-κB and MAPK), thus perpetuating the microglia inflammatory response. However, the precise mechanisms of the signaling interplay between RANK and TLRs are still to be fully revealed. RANK, receptor activator of nuclear factor-κB; RANKL, RANK ligand; OPG, osteoprotegerin; TRAF, TNF receptor-associated factor; IL, interleukin; TNF, tumor necrosis factor; TGF, transforming growth factor; IGF, insulin-like growth factor; CCL, CC-chemokine ligand; TRAIL, TNF-related apoptosis-inducing ligand; TLR, Toll-like receptor; NF-κB, nuclear factor-κB; IκB, inhibitor of NF-κB; PI3K/AKT, phosphatidylinositol 3-kinase/protein kinase B; MAPK, mitogen-activated protein kinase; AP-1, activator protein 1; NFATc1, nuclear factor of activated T cells, cytoplasmic 1.

In the physiological conditions, microglial cells exert two main functions: 1. Immune surveillance, and 2. Synaptic pruning. They also maintain neuronal homeostasis through the release of BDNF and neuronal growth factor (NGF) (36, 37). Moreover, they act as an innate immunity sentinel in the brain and present the first line of defense upon activation by brain tissue injury and infection. In such conditions, microglia upregulates expression of major histocompatibility complex (MHC) and costimulatory molecules, and enhances chemotaxis and recruitment of immune cells (36, 38, 39). Depending on the nature of the neuroinflammatory event, microglia may translate from physiological (termed “resting”) to pathological (termed “activated”) state, and orchestrate subsequent cellular events in favor of either pro-inflammatory or anti-inflammatory effector arm (Figure 2).

*In vitro* activated microglia polarizes into either pro-inflammatory (producing inflammatory cytokines, chemokines, and other mediators) or anti-inflammatory (mediating phagocytosis of tissue debris and regeneration) subtype, a concept adapted from polarization of peripheral tissue macrophages (40). Interleukin (IL)-1β, tumor necrosis factor (TNF)-α and interferon (IFN)-γ secreted from activated Th1 cells may enhance the inflammatory neurotoxic response and activate microglia to secret IL-1β, TNF-α, IL-6, CC-chemokine ligand 2 (CCL2), nitric oxide, and IL-12p70 (41–44). This triggers immune cell infiltration across BBB and neuronal damage. In contrast, IL-4 and IL-13 secreted by Th2 and Tregs direct microglia toward production of IL-4, IL-10, transforming growth factor (TGF)-β and insulin-like growth factor (IGF)-1. These mediators polarize the immune response toward neuroprotection and reparation, initiating the resolution of inflammation and secretion of neurotrophic factors (45–47).

Under physiological conditions microglial morphology shows branched processes oriented radially to a small elliptical soma, facilitating important functions in the refinement of synaptic networks, production of neurothropic factors, and removal of cell debris. Although polarization has not been clearly confirmed in an *in vivo* context, evidence shows that microglia undergo dynamical and temporal phenotypical and functional changes in response to brain injury. In diseased tissue microglia have enlarged cell bodies with fewer and shorter processes, mainly serving as phagocytic and innate immune cells (26, 40, 44).

Microglia also expresses Toll-like receptors (TLRs) which serve as pathogen recognition receptors (48). RANK expressed by both microglia and monocytes/macrophages, counteract TLR3- and TLR4-mediated signals. It has been shown that RANKL pre-treatment of microglial cells decreased the ability of TLR3/TLR4 pathway to induce the expression of inflammatory markers, such as inducible nitric oxide synthase (iNOS) and cyclo-oxygenase (COX) 2 (Figure 2). On the other hand, activated TLR4 suppresses the expression of RANK and potentiates the expression of TLR3, which in turn, amplifies pro-inflammatory signaling through a positive feedback loop. Since RANKL can decrease the pro-inflammatory effects of TLR agonists, RANKL/RANK signaling is thought to be neuroprotective in TLR-mediated neuroinflammation (28). However, the role of OPG remains controversial. It is possible that microglia and macrophages upregulate OPG production in order to neutralize the inhibitory effect of RANKL on overactive microglia. In parallel, increased secretion of OPG may have protective effect, by blocking the pro-apoptotic signaling of TNF-related apoptosis-inducing ligand (TRAIL) in microglia (28, 35, 49).

## RRO Axis in Different Pathological Conditions

The RRO axis has been studied in both, a variety of experimental animal models, and humans with different pathological conditions. These studies suggest that RRO triad may be a novel target for diagnostic and therapeutic approaches. Some research groups have developed RRO axis modifiers applicable to neurological setting and are working toward their application in clinic, some of these will be discussed below.

### RRO Axis and Brain Ischemia

Recent studies of the RANKL/RANK/OPG signaling pathway indicate its involvement in ischemic brain injury in particular in microglial activation. The relevance of microglia in ischemic injury is highlighted by its contribution to the reparative processes during the latter stages of CNS damage (50, 51).

In their resting state, microglial cells express negligible amounts of RRO mediators. In the active state and in the presence of TLR4 ligands, microglia up-regulate membrane expression of RANK and, simultaneously, enhance autocrine and paracrine secretion of sRANKL (48). Enhancement of RRO signaling decreases microglial activation by counteracting the TLR4 receptor pathway (48, 49, 52). Microglia at peri-infarction sites overexpress both, RANK and RANKL, which in turn, reduces ischaemic injury (49). *In vitro* studies using recombinant (r)RANKL confirmed this neuroprotective effect. However, it was seen only in mixed neuron/glia cell cultures, but not in cell cultures comprised only of cortical neurons. In peri-infarction areas, OPG is also overexpressed, but its exact cellular source is still under debate (35). Based on the data obtained through the *in vitro* studies, it is postulated that neurons and oligodendrocytes secrete OPG under ischaemic stress, and this secretion invokes damage to brain tissue due to its pro-inflammatory effect. OPG can even have the neurotoxic effect when overexpressed in hypoxic neurons, due to inhibition of RANK-signaling pathway in microglial cells (35).

In an experimental model of stroke (middle cerebral artery occlusion, MCAO), OPG deficient mice were found to have reduced infarction volume compared to wild-type (WT) controls. Administration of anti-RANKL neutralizing antibody increased the infarction volume in both OPG –/– and WT mice, although the effect was less pronounced in WT than in OPG –/– mice. These data imply that RANKL/RANK signaling has a protective role in ischaemic brain injury, whereas OPG exterts an opposite effect (49).

Based on the available data, it is proposed that blockade of TLR signal would reduce the extent of post-ischaemic brain injury. In such context, activation of the RANKL/RANK axis could be considered as an “anti-TLR” agent (Figure 2). However, systemic administration of rRANKL is limited in its clinical utility, as it would lead to clinically unacceptable osteoclast activation and osteoporosis in post-stroke patients (52). Recently, a recombinant anti-TLR agent, microglial-healing peptide (MHP)1, has been developed, and its structure is based upon the structure of the RANKL molecule. In the transient MCAO model, ischaemic injury was reduced by MHP1 injection either intra-cerebroventricularly at 4 h post-ischemia or intravenously 4/6 h post-ischaemia. Additionally, MHP1-AcN, a MHP1 derivative, can effectively attenuate tissue plasminogen activator-induced hemorrhage formation (53, 54). The only human study done on subject of brain ischemia and RRO axis showed that in Italian population, genetic variability of OPG gene (T245G, T950C, and G1181C polymorphisms) acts as an independent risk factor for ischemic stroke (55, 56). Some other studies that researched OPG levels in stroke patients were excluded from the review as they mainly focused on carotid calcifications and metabolism of calcium, which was not directly related to brain (stroke in these patients was rather the consequence of peripheral events).

These results collectively suggest that blockade of the RRO axis may lead to overstimulation of microglial cells, and in turn may exacerbate neural damage following tissue injury. In contrast, activation of RANK-signaling cascade ameliorates the unwanted inflammatory response, thus protecting the brain tissue from further damage.

### RRO Axis and Multiple Sclerosis

Autoimmune-mediated neuroinflammatory processes damage the integrity of the BBB and this results in a loss of immune privilege (57, 58). Besides being the structural part of the vascular wall, BBB functionally includes the Virchow-Robin's space, where the perivascular macrophages, constantly replenished by blood-born monocytes, juxtapose tunica media and contribute to its impermeability. The BBB is absent at certain sites, including the choroid plexus, retina and circumventricular organs. Immune cells recruited by CCL20 produced within the choroid plexus enter CNS and act within an immune surveillance capacity (57–59). However, in the presence of CNS inflammation immune privilege is lost, the BBB is compromised, and circulating immune cells can enter the CNS in an unregulated manner (60, 61). Intrathecal T cells infiltrate the brain parenchyma following their activation by specific brain tissue-resident antigen-presenting cells, such as microglia, astrocytes and dendritic cells (59, 60, 62). Evidence from murine models and human diseases has shown that activated T cells produce RANKL, especially in chronic inflammatory conditions (20, 21, 63, 64). A number of studies have sought to elucidate the underlying immunological mechanisms of RANKL expression and autoimmune inflammation in the brain.

Autoimmune disorders of the CNS, such as multiple sclerosis (MS), are associated with myelin sheath damage mainly caused by aberrant T cell response. The RRO axis has been found to potentially play a role in murine experimental autoimmune encephalomyelitis (EAE) as a model of human MS (59, 65, 66). In EAE, production of RANKL by T cells induces secretion of CCL20 in astrocytes. RANKL expression was found to be associated with trafficking of pathogenic T cells within brain parenchyma, particularly Th17 subset. RANKL inhibition exerted a reduction of T cell infiltration and a significant protective effect in murine EAE, and thus offers a potential therapeutic target (65, 67).

In relation to possible biomarkers, the concentrations of RANKL and OPG are found to be higher in the blood of MS patients. Primary progressive (PP)-MS is in particular associated with the highest levels of RANKL in parallel with other inflammatory makers (C-reactive protein [CRP], IL-1β, TNF-α) (68). Another study showed higher levels of RANKL in patients with relapsing-remitting (RR)-, PP- and secondary progressive (SP)- MS compared to healthy controls, and confirmed that RANKL levels were the highest in PP-MS (69). These studies focused only on the peripheral blood and did not examine the intrathecal expression of RRO mediators. Systemic higher levels of RANKL may reflect the disease pathogenesis, but as well induce additional effects including promotion of osteoclastogenesis, release of calcium from bone and peripheral activation of dendritic cells.

Through the parallel profiling of peripheral blood and cerebrospinal fluid (CSF), our group was the first to compare the levels of RANKL and OPG in control subjects and MS patients at clinical onset (70). It is important to stress that these naïve patients were excluded from the use of any immunomodulatory or immunosuppressive agents known to interfere with the RRO axis (71). CSF levels of OPG were decreased in patients with MS at clinical onset in comparison to healthy controls, together with higher sRANKL/OPG ratio, while there was no difference between groups in RANKL and OPG expression in peripheral blood. These data indicate the initial RRO dysregulation at intrathecal sites already at the outset of the disease. Additional analysis of plasma samples and peripheral blood mononuclear cells from patients with RR-MS revealed upregulation of chemokines CCL2 and CXCL12 as well as increase in sRANKL and sRANKL/OPG ratio with the disease progression (70). Another clinical study on MS patients found higher OPG and lower RANKL levels in serum of patients with RR-MS compared to those with SP-MS. However, chronic forms of MS are often associated with immunosuppressive treatment and immobility both of which affect the RRO axis, therefore it is difficult to exclude these as potential confounders (71, 72). Studies on MS patients during glatiramer-acetate (GA) or interferon (IFN)-β treatment showed, among other molecules, modulation of OPG levels (73, 74), supporting our claim about immunomodulatory treatment affecting RRO axis.

In conclusion, RRO axis has potential as both a disease biomarker and therapeutic target in certain autoimmune diseases of the CNS such as MS.

### RRO Axis in Brain Tumors

Primary CNS tumors account for a significant proportion of morbidity and mortality up to 29.9 per 100,000 persons (75, 76). Several studies have dealt with the RRO axis role in the malignant processes of different types of brain tumors.

Glioma cell lines and murine glioblastoma multiforme (GBM) models have demonstrated that GBM has employed immune escape mechanism that involve secretion of CCL20 and OPG, which acts directly and indirectly on microglia cells triggering their production of CCL2. This chemokine gradient attracts CCR4-expressing Tregs and CCR2-expressing monocytic myeloid-derived suppressor cells to the site of GBM, which in turn reduces the inflammatory response to tumor cells (77–80). One *in vivo* xenograft study, in which human glioma cells were stereotactically injected into mouse brain, revealed that GBM cells expressing high level of endogenous RANKL resulted in a more invasive tumor form compared to GBM cells expressing relatively low endogenous RANKL. Furthermore, the number of activated astrocytes was markedly increased in the periphery of RANKL-abundant invasive tumors, suggesting that RANKL is able to activate astrocytes through NF-κB signaling. These astrocytes have been shown to secrete various factors that regulate glioma cell invasion (81).

RANKL expression has also been examined in pituitary adenoma cell lines, in the context of death receptor activation. The cytotoxic agent containing the fused tripeptide sequence Arg-Gly-Asp (RGD) (interacting with transmembrane integrins) and Fas ligand was used for targeted activation of receptor Fas in several adenoma cell lines. In parallel to the increase in caspase expression, administration of RGD-Fas ligand stimulated the expression of RANKL, indicating that the RRO axis and its mediators are functionally linked to pro-apoptotic members of the TNF superfamily (82, 83).

### RRO Axis in Other Conditions Related to Brain

The RRO axis may be implicated in neurological conditions not directly related to ischaemic injury, neuroinflammation or tumorigenesis, indicating a general biological role within the CNS. A recent study of prednisolone-induced neurotoxicity in rat brain demonstrated that it is associated with the attenuation of RRO signaling through modulation of OPG and RANK expression (84). This study proposed that prednisolone-induced neurotoxicity is mediated by disturbances of the vitamin D3 endocrine signal. In contrast, vitamin D3 treatment mediates a critical neuroprotective role through reduced OPG expression and increased RANKL expression, resulting in enhanced RANK signaling (84, 85). It appears that activation of the RRO axis and greater bioavailability of vitamin D3 in the CNS decrease activation of pro-inflammatory microglia. Thus, the interplay of vitamin D3 and RRO signal may prevent autoimmune response as well as influence degenerative diseases such as Alzheimer's disease.

There is also evidence that RANKL reduces food intake and causes weight loss via modulating the hypothalamic neuropeptide Y(NPY)/cocaine- and amphetamine-regulated transcript (CART) peptide pathways (86, 87). RANK deletion from NPY neurons may downregulate NPY mRNA expression in hypothalamus, indicating that RANK signaling regulates the balance between bone mass and body weight by modulating NPY levels (87).

Three studies suggest the involvement of RRO axis in thermoregulation. It seems that RANKL may activate brain regions involved in thermoregulation and induce fever via COX2-prostaglandin E2/EP3 receptor pathway in neurons and astrocytes. RANK mutation in both rodents and humans results in an abrogated fever response compared to controls (88–90).

## Conclusion

The increasing evidence is accumulated that RRO axis has an important role in neural tissue pathophysiology and could be a common functional link between different inflammatory processes within the CNS.

The elements of RRO axis in CNS include immune cells, predominantly microglia, but also other resident and infiltrating macrophages, mediating homeostatic and neuroinflammatory mechanisms. The expression in neurons and oligodendrocytes is mainly confined to differentiation and apoptotic processes. RRO axis tunes the neuroinflammatory response, highly depending on the tissue context. Research into RRO signaling has demonstrated apparent contradictory effects and suggests that RRO signaling is beneficial in ischemic lesions and deleterious in autoimmune reactions. We propose that this paradox may be explained by the stimulation of different microglial subtypes and the induction of diverse downstream effector functions (Figure 2). However, precise regulation of microglia activity *in vivo* by RRO mediators and their functional interactions with other cytokines and growth factors still need to be fully elucidated.

To date most studies involving RRO mediators in human samples have focused on the peripheral blood biomarkers. Future research is needed to identify their specific effects within brain tissue and to develop novel agents that can modulate RRO signaling. Several such modulators are presently in clinical use for different conditions, including RANKL agonists as well as OPG-mediated axial antagonists. We believe that modulation of the RRO axis may complement current treatment strategies for certain neurological disorders providing novel therapeutic and diagnostic targets.

## Data Availability Statement

The original contributions presented in the study are included in the article/supplementary materials, further inquiries can be directed to the corresponding author/s.

## Author Contributions

AG, NO'M, DG, and SG: conception and design of the study. AG, NO'M, NK, DG, and SG: agreement to be accountable for all aspects of the work, final approval of the version to be submitted, critical revision of the manuscript for important intellectual content, drafting of the manuscript, analysis, and interpretation of the data. All authors contributed to the article and approved the submitted version.

## Conflict of Interest

The authors declare that the research was conducted in the absence of any commercial or financial relationships that could be construed as a potential conflict of interest.
