# Supplementary material for: RANK/RANKL/OPG Signaling in the Brain: A Systematic Review of the Literature
Source: Front Neurol. 2020 Nov 19;11:590480. doi: 10.3389/fneur.2020.590480 (PMC7710989; doi:10.3389/fneur.2020.590480)
Supplement: Supplementary file 1 [file Data_Sheet_1.doc]

**Supplemental material 1**

**Search methods for identification of studies**

We conducted a comprehensive search (July 2019).

PICO model used:

P – CNS

I – RRO axis

C - /

O – CNS functioning and corresponding pathologies.

We developed detailed search strategies, based on the strategy for MEDLINE but revised appropriately for each resource.

We searched the following databases:

1. Cochrane Central Register of Controlled Trials (CENTRAL/Cochrane Library; via

Ovid)
2. MEDLINE (via Ovid)
3. Scopus
4. Web of Science Core Collection (via Web of Science)
5. Current Contents (via Web of Science)
6. SciELO Citation Index (via Web of Science)
7. KCI-Korean Journal Database (via Web of Science)
8. Russian Science Citation Index (via Web of Science)

9. BIOSIS Citation Index (via Web of Science)

10. Data Citation Index (via Web of Science)

We searched additional resources including:
1. ProQuest Dissertations & Theses Global
2. OpenGrey
3. African Index Medicus
4. IndMED
5. ClinicalTrials.gov
6. WHO International Clinical Trials Registry Platform (WHO ICTRP)
7. CenterWatch

Search strategy for MEDLINE/Ovid

1. RANK Ligand/
2. (RANK Ligand OR RANKL OR RANKL protein OR OPGL protein OR osteoclast differentiation factor OR osteoprotegerin ligand OR tumor necrosis factor ligand superfamily member 11 OR TNF superfamily member 11 OR tumor necrosis factor related activation induced cytokine OR antigen CD254 OR TRANCE protein).tw
3. ((receptor activator of nuclear factor OR receptor activator of NF) adj3 ligand).tw
4. 1 OR 2 OR 3
5. Receptor Activator of Nuclear Factor- kappa B/
6. (Receptor Activator of Nuclear Factor kappa Beta OR Receptor Activator of Nuclear Factor kappa B OR Receptor Activator of Nuclear Factor kappaB OR RANK Protein OR TRANCE R OR TRANCE Receptor OR Receptor Activator of NF kappaB OR Receptor Activator of NF kappa B OR Receptor Activator of NF kappa beta OR Receptors, Tumor Necrosis Factor, Member 11a OR Tumor Necrosis Factor Receptor Superfamily, Member 11a OR NF Kappa B Receptor Activator OR CD265 Antigen OR TNFRSF11A Protein).tw
7. 5 OR 6
8. Osteoprotegerin/
9. (OPG OR osteoprotegerin OR osteoclastogenesis inhibitory factor OR receptors, tumor necrosis factor, member 11b OR follicular dendritic cell derived receptor 1 OR tumor necrosis factor receptor superfamily, member 11b OR FDCR 1 protein OR OCIF protein OR tumor necrosis factor receptor 11b).tw
10. 8 OR 9
11. 4 OR 7 OR 10
12. exp Brain/
13. brain.tw
14. 12 OR 13
15. exp Spinal Cord/
16. (spinal adj1 cord$).tw
17. (medulla adj1 spinali$).tw
18. 15 OR 16 OR 17
19. exp Central Nervous System/
20. (central nervous system$ OR system$ central nervous).tw
21. (cerebrospinal adj1 axi$).tw
22. 19 OR 20 OR 21
23. 14 OR 18 OR 22
24. 11 AND 23
25. exp Multiple Sclerosis/
26. (MS OR multiple sclerosis acute fulminating).tw
27. (multiple adj1 sclerosis).tw
28. (sclerosis adj1 disseminated)).tw
29. OR/25-28
30. 11 AND 29
31. exp Brain Neoplasms/
32. (brain adj1 (neoplasm$ OR tumor$ OR cancer$)).tw
33. (intracranial adj1 neoplasm$).tw
34. (brain malignant neoplasm$ OR malignant neoplasm$ brain OR brain neoplasm$ malignant OR malignant brain neoplasm$ OR brain tumor$ recurrent OR primary malignant brain tumor$ OR primary malignant brain neoplasm$ OR brain benign neoplasm$ OR benign neoplasm$ brain OR brain tumor$ primary OR primary brain tumor$ OR primary brain neoplasm$ OR neoplasm$ brain primary ).tw
35. OR/31-34
36. 11 AND 35
37. exp Stroke/
38. (stroke$ OR cerebrovascular accident$ OR cva$ OR brain vascular accident$ OR vascular accident$ brain OR apoplexy OR cerebrovascular accident$ acute OR acute cerebrovascular accident$).tw
39. (cerebrovascular adj1 (apoplexy OR stroke$)).tw
40. (cerebral adj1 stroke$).tw
41. (stroke$ adj1 acute).tw
42. OR/37-41
43. 11 AND 42
44. exp Dementia/
45. (dementia$ OR amentia$ OR senile paranoid dementia$ OR primary senile degenerative dementia). tw
46. (AIDS dementia complex OR alzheimer$ disease OR alzheimer$ syndrome OR aphasia$ primary progressive OR Mesulam$ syndrome OR Creutzfeldt Jacob disease$ OR Creutzfeldt Jakob Syndrome OR familial Creutzfeldt-Jakob disease$ OR diffuse neurofibrillary tangles with calcification OR Kosaka Shibayama disease OR frontotemporal lobar degeneration$ OR Huntington$ disease OR Kluver Bucy Syndrome OR Lewy Body dementia OR Lewy Body disease).tw
47. (familial adj1 dementia$).tw
48. (dementia$ adj1 senile).tw
49. (HIV adj1 dementia$).tw
50. (encephalopath$ adj1 AIDS).tw
51. (alzheimer$ adj1 (sclerosis OR dementia$)).tw
52. (dementia$ adj1 vascular).tw
53. (arteriosclerotic adj1 dementia$).tw
54. (Huntington adj1 chorea).tw
55. OR/44-54
56. 11 AND 55
57. 24 OR 30 OR 36 OR 43 OR 56
