# Supplementary material for: RANK/RANKL/OPG Signaling in the Brain: A Systematic Review of the Literature
Source: Front Neurol. 2020 Nov 19;11:590480. doi: 10.3389/fneur.2020.590480 (PMC7710989; doi:10.3389/fneur.2020.590480)
Supplement: Supplementary file 2 [file Data_Sheet_2.docx]

Supplemental material 2

Final selected studies
